# PROTOCOL: Development evaluations in India 2000–2018: A country impact evaluation map

**DOI:** 10.1002/cl2.1122

**Published:** 2020-10-06

**Authors:** Ashrita Saran, Eti Rajwar, Bhumika T. V., Divya S. Patil, Howard White

**Affiliations:** ^1^ Campbell Collaboration New Delhi India; ^2^ Public Health Evidence South Asia, Prasanna School of Public Health, Manipal Academy of Higher Education Manipal Karnataka India

## 1 BACKGROUND

### 1.1 The problem, condition, or issue

Development evaluation refers to evaluation of development programs, which are defined as any social or economic programs aimed at community development. These are generally funded by the government, aid agencies, nongovernmental organizations, foundations, or firms under corporate social responsibility. Invariably, most of them require that their programs be evaluated to understand if the intervention succeeded or failed (Bamberger, 2000).

Public development programs have been an important component of the development policy of the Government of India. The first 5 year plan deemed that systematic evaluation should become a normal administrative practice in all spheres of public activity and for this the Planning Commission (PC) began developing the evaluation techniques by establishing Program Evaluation Organisation for independent evaluations of community projects and other intensive area development programs (Chandrasekar, 2015). PC of India which was later dissolved and new organization named National Institution for Transforming India (NITI Aayog) was created on January 1st, 2015. The NITI Aayog is responsible for preparing the policy framework, designing the public programs and also monitors and evaluates the outcomes achieved by different programs (NITI Aayog, 2018).

There has been and increasing trend of impact evaluations in low and middle income countries over the years. The largest share (34.4%) of studies is conducted in Sub‐Saharan Africa followed by Latin America and Caribbean with 24.7% of the total studies, while South Asia ranks third at 17%. Nearly half of South Asian studies are conducted in India (Sabet & Brown, 2018). Though the numbers of impact evaluations is growing there is no central repository for easy access or to identify sectors with evident gaps.

Also, in a diverse country like India implementation and flawless evaluation of all the public development programs is a great challenge. Timely evaluations of such programs are often pushed by the policy makers and the program implementers to increase the accountability and for improvement of the program (Malhotra, 2014).

Despite, effective measures taken by the union and state governments there are evident gaps present in evaluation of these public development programs. Identification of these gaps will help in making the programs more effective and will eventually support in improving the development indicators of the country.

Evaluations of public programs also contributes toward cost cutting and cost‐effectiveness as it gives information about what works and what does not. This is essential because of the rise in public expenditure and economy of India. Appropriate monitoring and evaluation of a program helps to identify the challenges in implementation of a particular program and provides an evidence base for decisions pertaining to public resource allocation; leading to more effective public policy development (Malhotra, 2014).

Over the past decades demand for program evaluations have increased greatly as emanating from civil society, enforced by the Supreme Court and Comptroller and Auditor General and government ministries, especially at the level of central government. Also donors, who although having a relatively small role in India, also support evaluations. Multilateral agencies (the World Bank, the Asian Development Bank, and the United Nations system agencies) and five bilateral donors present in India (the United States, the United Kingdom, Germany, France, and Japan) support evaluation of programs to which they make a financial contribution.

In an effort to align the “Strategy for New India” with India's commitment to the United Nations' Sustainable Development Goals (SDGs), NITI Ayog with extensive consultations and inputs published “Strategy for New India @ 75.” It has identified 41 different areas that require either a sharper focus on implementing the flagship schemes already in place or a new design and initiative to achieve India's true potential.

### 1.2 Scope of the evaluation map

The proposed country evidence and gap map will include all published impact evaluations of development interventions implemented in India from 2000 to 2018. Development is broadly defined to cover all interventions intended to either directly or indirectly improve the well‐being of the people of India. Technical studies (e.g., clinical trials of new drugs or testing new agricultural inputs) are excluded, though efficacy and effectiveness evaluations of the adoption and use of these technologies under field conditions are included.

### 1.3 Why it is important to develop the evaluation map

There are close to 400 impact evaluations identified in India through initial search of international initiative for impact evaluations (3ie). But what we do not know is the spread of these impact evaluations across the intervention sectors and SDG. Several important questions have not been adequately addressed. For example, what type of evidence is needed, and what are realistic expectations for outcomes and effectiveness research? Knowledge production takes place across several sectors (health, social welfare, and education), focuses on various populations (different ages, ethnicities, or with different needs), and involves rather diverse methodical approaches (e.g., systematic reviews, primary studies of different designs, etc.).

A mapping of the existing knowledge base is therefore required to provide a comprehensive overview of existing knowledge in this area and enable the purposeful and targeted commissioning of future evaluations, tailored to the most eminent needs for knowledge and guidance. This ambition could be fulfilled by proposed Evidence and Gap Maps (EGM).

### 1.4 Existing EGMs and/or relevant systematic reviews

To our knowledge this will be the first country evaluation map for development impact evaluations for India. Though similar maps are ongoing in Uganda and Kenya.

## 2 OBJECTIVES

The primary objective of the India country evaluation map is to make the existing impact evaluations accessible to the users, and to support evidence‐informed decision‐making across government, development partners, and civil society.

Secondary objectives are to

(i) Identify gaps in available evidence, and clusters of evidence suitable for systematic reviews
(ii) Raise awareness of the use of evidence in policy and practice,
(iii) Initiate discussions around impact evaluation trends in India, and systemic effects on evaluation transparency and quality, and

The research question being addressed to support these objectives is: what is the extent of the evidence base of Impact evaluations of development programs in India?

## 3 METHODOLOGY

### 3.1 Defining country evaluation maps

Saran and White (2018) defined EGMs as “a systematic visual presentation of the availability of relevant evidence of effects for a particular policy domain.” The evidence is identified by a search following a prespecified, published search protocol. The map may be accompanied by a descriptive report to summarize the evidence for stakeholders such as researchers, research commissioners, policy makers, and practitioners. Evidence and gap maps summarize what evidence there is, not what the evidence says.”

Similar to EGMs, country evaluation maps systematically search for relevant systematic reviews and impact evaluations based on a prespecified, published search protocol, and provide graphical representation of available evidence. The scope of country evaluation map, however, is larger than typical EGM as the former will include all policy domains that will affect the welfare of the people of the country, and include only Impact evaluations.

### 3.2 Evaluation map framework

The framework has been developed through the following process:

Stage 1: Initial framework is constructed through consultation of strategy and policy documents as identified from NITI Ayog strategic goals for a new India by 2022.

Stage 2: Piloting of the framework with 10–20 included studies. The framework was finalized based on pilot coding of 10–20 studies. Any subsequent changes to the framework will be recorded as deviations from protocol.

### 3.3 Population

The relevant population for this map is both the people (including refugees) and institutions of India. Population subgroups will be: rural/urban, poor, people with disabilities, children, youth, aged, ethnic minorities, refugees, conflict‐affected persons.

### 3.4 Intervention

The map will cover all development interventions. The categorization of the interventions follows the categories in NITI Ayog's vision 2022. These are shown in Table 1.

**Table 1:** Intervention categories and subcategories

Intervention categories	Description	Intervention subcategories
Drivers	Drivers focus on the engines of economic performance such as on growth and employment, doubling of farmers' incomes; upgrading the science, technology and innovation ecosystem; and promoting sectors like tourism	Growth and employment
		Agriculture productivity and farmer income
		Strengthening manufacturing sector
		Science technology and Innovation
		Tourism
Infrastructure	Infrastructure deals with the physical foundations of growth which are crucial to enhancing the competitiveness of Indian business as also ensuring the citizens' ease of living	Energy
		Transport
		Urban and rural development
		Environment and water resources
Inclusions	Inclusion deals with the urgent task of investing in the capabilities of all of India's citizens. The three themes in this section revolve around the dimensions of health, education and mainstreaming of traditionally marginalized sections of the population	Education
		Health
		Nutrition
		Gender equality
Governance	Governance delves deep into how the governance structures can be streamlined and processes optimized to achieve better developmental outcomes	Balanced regional development
		Legal, judicial and police reforms
		Civil service reforms
		City governance and use of land resources
		Data‐led governance

#### 3.4.1 Outcomes

The outcomes are based on the SDGs (Table 2). The Government of India recently committed to developing a roadmap to achieve the SDGs, so the country evidence map will be a useful tool in that work (Table 3).

**Table 2:** Outcomes

Economic development (including poverty and employment) *SDGS 1 and 8*
Sustainable agriculture *SDG 2*
Nutrition *SDG 2*
Health and well‐being *SDG 3*
Education *SDG 4*
Gender *SDG 5*
Water and sanitation *SDG 6*
Energy, industry and infrastructure provision *SDGs 7 and 9*
Urban development *SDG 11*
Environmental sustainability *SDGs 12, 13, 14 and 15*
Peace and justice *SDG 16*
Global partnerships *SDG 17*
Inequality *SDG 10*

Abbreviation: SDG, sustainable development goal.

**Table 3:** Summary of PICOS

Population	Indian citizens or people resident in India (including refugees)
Intervention	All interventions to increase the welfare of Indian citizens or people resident in India (including refugees) either direct or indirectly
	Exclude: studies which test a technology
Comparison	Not applicable
Outcomes	All outcomes (as captured by sustainable development goals), including intermediary outcomes
Studies	The study must be an impact evaluation of a socio‐economic development intervention (so clinical trials are excluded)

#### 3.4.2 Criteria for including and excluding studies

##### Types of study designs

Only Impact evaluations will be included. Definition of Impact evaluation as described by Scriven's (1967):

Impact evaluations: Impact evaluations are defined as intervention evaluations or field experiments that use quantitative approaches applied to experimental or observational data to measure the effect of an intervention relative to a counterfactual representing what would have happened to the same group in absence of that intervention. Impact evaluations may also test different intervention designs.

We will include studies that assess the effects of interventions using experimental and quasiexperimental study designs that allow for causal inference. Specifically, we will include:

1. Studies where participants are randomly assigned to treatment and comparison group
2. Studies where assignment to treatment and comparison group is based on other known allocation rules, including a threshold on a continuous variable (regression discontinuity designs). We will include

Regression Discontinuity Designs, Fixed Effect Estimation, Instrumental Variable, Propensity Score Matching and Difference‐in‐Difference.

Before‐versus‐after studies with no comparison group will not be included. Impact evaluation designs will be coded as randomized control trials and other impact evaluations.

Since the inclusion criteria are for impact evaluations of development interventions in India, systematic reviews are not included in this map.

##### Types of settings

The map will include evaluations studies undertaken in India regardless of setting.

##### Status of studies

We will include both completed and ongoing impact evaluations; to capture the latter, we will include prospective study records in trial registries or protocols when available.

##### Search methods for identification of studies

###### Electronic searches

Relevant impact evaluations will be identified through the main impact evaluation databases. The searches will include studies published from inception to the present.

**Table:** 

Organization	Searchable by	Overlap with IE databases
International Initiative for Impact Evaluation: Find Evidence	Keyword “Impact evaluation”, country “India”	DIME, Poverty Impact Evaluations Database, JPAL, IPA, CEGA, OECD DAC DeREC
World Bank: Development Impact Evaluation Initiative (DIME)	Keyword “Impact evaluation”, country “India”	Poverty Impact Evaluations Database, 3ie
World Bank: Poverty Impact Evaluations Database	Keyword “Impact evaluation”, country “India”	DIME, 3ie
Abdul Latif Jameel Poverty Action Lab: Evaluations	Keyword “Impact evaluation”, country “India”	3ie, IPA
Innovations for Poverty Action (IPA): Search Projects	Keyword “Impact evaluation”, country “India”	JPAL, 3ie
University of California Center for Effective Global Action (CEGA): Research Projects	Keyword “Impact evaluation”, country “India”	3ie, Jpal, and IPA
Asian Development Bank: Independent Evaluation's Evaluation Resources	Keyword “Impact evaluation”, country “India”	OECD DAC DeRec
Poverty and Economic Policy Research Network (PEP): Project List	keyword, sector or theme, country or region	–
World Bank: Independent Evaluation Group's “Impact Evaluations”	–	OECD DAC DeRec
Inter‐American Development Bank: Office of Evaluation and Oversight	Keyword “Impact evaluation”, country “India”	OECD DAC DeRec
USAID: Development Experience Clearinghouse	Keyword “Impact evaluation”, country “India”	OECD DAC DeRec
OECD Development Assistance Committee: Evaluation Resource Center (DEReC)	Keyword “Impact evaluation”, country “India”	DIME, Poverty Impact Evaluations Databse, AsDB, IDB, AfDB, EBRD, 3ie
African Development Bank: Evaluation Reports	Country “India”	
DFID: Evaluation Reports	Country “India”	
Millennium Challenge Corporation: Evaluation Catalog		

Where the database allows a data range then the search is for 2000. If that is not possible then records pre‐2000 are removed by manual screening.

###### Other sources

We will search Google Scholar using the very general search string “India Impact evaluation” (not an exact string), applying the ≥2,000 date range to identify additional gray literature. We will also search other sources as will be identified through expert consultations and as submitted as a result of dissemination of the Evaluation Map.

####### Impact evaluation and study terms

((match* adj3 (propensity or coarsened or covariate)) or “propensity score” or (“difference in difference*” or “difference‐in‐difference*” or “differences in difference*” or “differences‐in‐difference*” or “double difference*”) or (“quasi‐experimental” or “quasi experimental” or “quasi‐experiment” or “quasi experiment”) or ((estimator or counterfactual) and evaluation*) or (“instrumental variable*” or (IV adj2 (estimation or approach))) or “regression discontinuity”) ti,ab,kw

(((experiment or experimental) adj2 (design or study or research or evaluation or evidence)) or (random* adj4 (trial or assignment or treatment or control or intervention* or allocat*))) ti,ab,kw

Randomized Controlled Trial/or random allocation/or Propensity Score/or Models, Econometric/or Quasi‐Experimental Studies/MeSH

Program Evaluation/or Evaluation Studies/MeSH

((impact adj2 (evaluat* or assess* or analy* or estimat* or measure)) or (effectiveness adj2 (evaluat* or assess* or analy* or estimat* or measure))). ti,ab,kw.

(“program* evaluation” or “project evaluation” or “evaluation research” or “program* effectiveness”) ti,ab,kw

### 3.5 Screening and selection of studies

The screening and coding tools are devised by A. S., who is leading the study who has prepared and managed the search for several Campbell maps. The entire process is guided by Howard white who is leading and known development expert. The tool will be piloted by the team members. All the team members have previous experience of working on systematic reviews and are well versed with screening and coding methodology.

Titles and abstracts will be screened against the inclusion criteria and relevant records will be downloaded into the review management software EPPI reviewer. The initial screening of records will be conducted by several reviewers screening the records from different databases. At this stage we will be over‐inclusive to ensure relevant studies are not omitted because sufficient information is not reported in title or abstract. Two reviewers will then independently review abstracts that have been judged to be potentially relevant at the first stage in more detail to determine which papers should be retrieved and reviewed at full text.

Two reviewers will then independently assess full text studies for inclusion, with any disagreements determined by a third reviewer.

### 3.6 Data extraction, coding, and management

Coding will be carried out in EPPI reviewer by two independent reviewers with third resolving the conflict.

The studies shall be coded on the basis of the following information:

1. **Bibliographic Information**

**Table:** 

Title	
Author(s)	
Month	
Year	
Publication Type	
Abstract	
Journal title or Report Series	
URL	
Volume	
Publisher	
DOI	
Short title [First author (date)]	
Edition	
ISBN/ISSN	
Issue	
Institution	
Country	India
2. **Intervention categories and subcategories**

**Table:** 

Intervention categories	Intervention subcategories
Drivers	Growth and employment
	Agriculture productivity and farmer income
	Strengthening manufacturing sector
	Science technology and Innovation
	Tourism
Infrastructure	Energy
	Transport
	Urban and rural development
	Environment and water resources
Inclusions	Education
	Health
	Nutrition
	Gender equality
Governance	Balanced regional development
	Legal, judicial and police reforms
	Civil service reforms
	City governance and use of land resources
	Data‐led governance
3. **Outcomes**

**Table:** 

Economic development (including poverty and employment) *SDGS 1 and 8*
Sustainable agriculture *SDG 2*
Nutrition *SDG 2*
Health and well‐being *SDG 3*
Education *SDG 4*
Gender *SDG 5*
Water and sanitation *SDG 6*
Energy, industry and infrastructure provision *SDGs 7 and 9*
Urban development *SDG 11*
Environmental sustainability *SDGs 12, 13, 14 and 15*
Peace and justice *SDG 16*
Global partnerships *SDG 17*
Inequality *SDG 10*
4. **Filters and other**

**Table:** 

Population	Rural
	Urban
	People with disabilities
	Children
	Youth
	Aged
	Ethnic minorities
	Refugees
	Conflict‐affected persons
Impact evaluations	Randomised controlled trials
	Regression discontinuity designs
	Fixed effect estimation
	Instrumental variable
	Propensity score matching
	Difference‐in‐difference
Geographical coverage	National
	Northern Zonal Council
	Northern Eastern Council
	Eastern Zonal Council
	Central Zonal Council
	Western Zonal Council
	Southern Zonal Council
	Special Invitees to Southern Zonal Council
Funding agency	
Commissioning agency	
Authorship	All India authorship
	Indian lead author with non‐Indian authors
	Indian authors with non‐Indian lead
	No Indian authors

### 3.7 Quality appraisal

Critical appraisal will not be undertaken at this stage, but is considered for future editions of the map.

## 4 ANALYSIS AND PRESENTATION

### 4.1 Unit of analyses

Each entry in this EGM will either be an impact evaluation. The final EGM will identify the number of studies covered by the map in each sector or subsector. However, an impact evaluation may be entered multiple times depending on issues covered and study design employed. For multiple interventions, studies will be coded for the main intervention categories.

### 4.2 Planned analyses

The EGM will be supplemented by an EGM report that—based on tables and/or graphs—will descriptively summarize the number of studies included in the EGM and their distribution across different coding categories such as study type, geography, target populations, interventions, and outcomes. Each table/graph will be accompanied by brief narrative descriptions. The report will also discuss the potential use of the EGM and highlight its boundaries and limitations.

The report accompanying the map will provide tables representing the number of studies by

- The types of evaluations
- Sectors and subsectors
- SDG domains and subdomains
- Year
- Source
- Nationality of authors
- Geographic distributions

Cross tabulation of some of these variables will also be presented.

### 4.3 Presentation

The primary dimensions of the map will be the types of evaluation (in rows) and various SDG indicators and subcategories in the column. Filters will be provided for secondary dimensions such as rural, urban, people with disabilities, children, youth, aged, ethnic minorities, refugees, and conflict‐affected persons.

## 5 STAKEHOLDER ENGAGEMENT

This framework for the map will be through a consultation process. The initial framework was constructed through consultation of strategy and policy documents. The framework and later the draft and final reports will be discussed with the Advisory Group for their inputs and feedback.

## ROLES AND RESPONSIBILITIES

- Content: H. W. who has over 10 years of experience in working with international development.
- EGM methods: H. W. and A. S., who have coauthored four EGMs together. H. W. has assisted the development of Campbell collaboration guidelines and standards.
- Information retrieval: A. S. has drafted and designed the search strategy for all the Campbell EGM's.
- Coding and data extraction: All the authors have worked in conducting systematic reviews and will be responsible for the activity. Training and supervision on Eppi‐reviewer will be provided by A. S.

## SOURCES OF SUPPORT

This EGM has seed support from the Campbell Collaboration. Additional funding is being sought.

## DECLARATIONS OF INTEREST

None.

## PRELIMINARY TIMEFRAME

Protocol: February 2020

Draft map and report: December 2020

## FUNDING

This map does not have any funding.

## PLANS FOR UPDATING THE EGM

Currently no funding is available for this purpose. It will be sought once the map is published.
